# Insulin Oedema in Newly Diagnosed Type 1 Diabetes Mellitus

**DOI:** 10.4274/jcrpe.v2i1.46

**Published:** 2010-12-08

**Authors:** Veysel Nijat Baş, Semra Çetinkaya, Sebahat Yılmaz Ağladıoğlu, Havva Nur Peltek Kendirici, Hatice Bilgili, Nurdan Yıldırım, Zehra Aycan

**Affiliations:** 1 Dr. Sami Ulus Women Health, Children's Education and Research Hospital Section of Pediatric Endocrinology, Ankara, Turkey; +90 312 305 65 08+90 312 317 03 53veyselbas@yahoo.comDr. Sami Ulus Women Health, Children's Education and Research Hospital, Clinics of Pediatric Endocrinology, Ankara, Turkey

**Keywords:** type 1 diabetes

## Abstract

Despite the essential role of insulin in the management of patients with insulin deficiency, insulin use can lead to adverse effects such as hypoglycaemia and weight gain. Rarely, crucial fluid retention can occur with insulin therapy, resulting in an oedematous condition. Peripheral or generalised oedema is an extremely rare complication of insulin therapy in the absence of heart, liver or renal involvement. It has been reported in newly diagnosed type 1 diabetes, in poorly controlled type 2 diabetes following the initiation of insulin therapy, and in underweight patients on large doses of insulin. The oedema occurs shortly after the initiation of intensive insulin therapy. We describe two adolescent girls with newly diagnosed type 1 diabetes, who presented with oedema of the lower extremities approximately one week after the initiation of insulin treatment; other causes of oedema were excluded. Spontaneous recovery was observed in both patients.

**Conflict of interest:**None declared.

## INTRODUCTION

Insulin is essential in type 1 diabetes, including the emergency treatment of ketoacidosis. It may also be needed in type 2 diabetes for intercurrent events like surgery, infections, serious illness, trauma, pregnancy and for emergency treatment of hiperkalemia (1). While insulin has an essential role in the management of patients with insulin deficiency, its clinical use may lead to adverse effects such as hypoglycaemia and weight gain. The reason of weight gain associated with insulin treatment is poorly understood and has been attributed to increased vascular permeability and shifted renal sodium handling in addition to the anabolic effect of insulin. Rarely, crucial fluid retention can occur with insulin therapy. Peripheral or generalised oedema is an extraordinary complication of insulin therapy in the absence of heart, liver or renal involvement. It has been reported in newly diagnosed type 1 diabetes, in poorly controlled type 2 diabetes following the initiation of insulin therapy, and in underweight patients on large doses of insulin. The oedematous state develops briefly after the initiation of intensive insulin therapy (2, 3, 4). The pathophysiology remains vague, although the condition was first described as early as 1928 (3). Reported cases of insulin-induced oedema in childhood and adolescence are scarce (1, 5). The first pediatric report dates back to 1979 (6), and ten more cases have been reported since then (6, 7, 8, 9). Herein we describe insulin oedema in two adolescent girls with newly diagnosed diabetes.

## CASE 1

**Figure 1:**
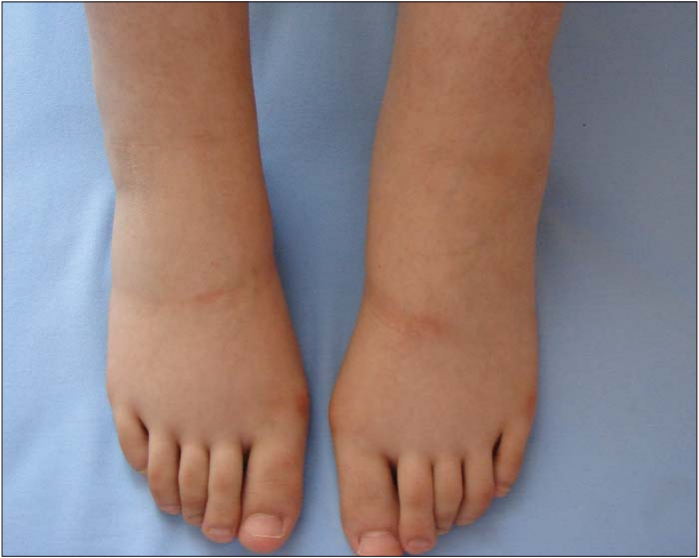
Lower extremity oedema 4 days after the initiation of insulin therapy in Case 1

A 14-year-old girl was admitted with a 1-month history of polyuria and polydipsia. A recent weight loss of 5 kg was also mentioned. No family history of diabetes was reported. Physical examination at the time of admission revealed a temperature of 36.70C, a pulse of 66 beats per minute, a respiratory rate of 24 per minute, and a blood pressure of 110/70 mm/Hg. The patient’s height was 161 cm (-0.01 SDS) and her weight was 40 kg (-2.85 SDS). Calculated body mass index was 15.6 kg/m^2^ (-2.7 SDS). Careful history excluded anorexia nervosa as a contributing condition to her weight loss. She was at pubertal Tanner stage 5. Laboratory investigations revealed a blood glucose level of 363 mg/dL, ketonuria with acidosis, an arterial blood pH of 7.2 and an elevated glycosylated haemoglobin A1c concentration of 12.1%. The patient was initially treated with an intravenous infusion of insulin and 2/3 isotonic saline in 5% dextrose. Mild bilateral pitting ankle oedema and discoloration of the skin developed over the ankles. The oedema deteriorated on the fourth day, after the initiation of regular insulin administered subcutaneously, at a dose of 1.2 units/kg/day (Figure 1). No evidence of heart, liver or kidney dysfunction was noted. Serum albumin levels decreased from 3.5 to 3.2 g/dL, without proteinuria. Chest X-ray and abdominal ultrasound findings were normal. Doppler ultrasound findings of the lower leg arteries and veins were normal. Ten days later the oedema had completely resolved without any specific treatment.

## CASE 2

**Figure 2:**
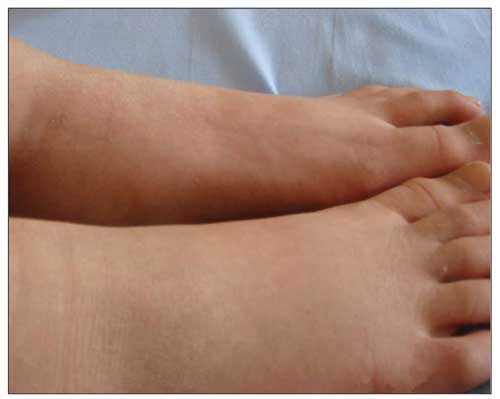
Lower extremity oedema 5 days after the initiation of insulin therapy in Case 2

An 11-year-old girl was admitted with an 8-month history of polyuria, polydipsia and a recent weight loss of 7 kg. No family history of diabetes was reported. Physical examination at the time of admission revealed a temperature of 370C, a pulse rate of 96 beats per minute, a respiratory rate of 28 per minute, and a blood pressure of 100/65 mm/Hg. Her height was 147 cm (-0.31 SDS) and her weight 30 kg (-1.75 SDS). Calculated body mass index was 13.8 (-1.9 SDS) kg/m2. Pubertal stage was evaluated as Tanner stage 3. Physical examination was normal except for clinical signs of mild dehydration. Laboratory investigations revealed a blood glucose level of 453 mg/dL, ketonuria with acidosis, an arterial blood pH of 7.1 and an elevated glycosylated haemoglobin A1c concentration of 13.9%. The patient was treated with 2/3 isotonic saline in %5 dexstrose and intravenous insulin infusion. On the fifth day of regular insulin administration, a non-tender, pitting oedema without skin discoloration developed over the ankles (Figure 2). No evidence of heart, liver or renal dysfunction was noted. Serum albumin levels remained stable. Chest X-ray and abdominal ultrasound findings were normal. Doppler ultrasound findings of the lower leg arteries and veins were normal. Seven days later the oedema disappeared completely without any treatment. 

Further investigations revealed positive anti-GAD (glutamic acid decarboxylase) and anti-islet cell antibodies in both patients.

## DISCUSSION

Insulin oedema is presumably an underreported physical finding. The severity of the peripheral oedema is variable, most cases being mild. Oedema has been accepted as an uncommon complication occurring after initiating or intensifying the insulin treatment (3). Leifer (2), in 1928, proposed that rapid retention of tissue fluid secondary to glycogen deposition was responsible for the pathophysiology of insulin oedema. In subsequent years, the direct antinatriuretic effect of insulin on the kidney was recognised and insulin oedema was attributed to alterations in renal electrolyte transport (10). Insulin-induced oedema was found to occur equally in adult men and women, but a clear female predominance was noted in younger ages (3, 6). Both our patients were also females. In addition, the reported pediatric cases were all recently diagnosed patients with type 1 diabetes, similar to our patients. Intensive fluid resuscitation in an insulin-deficient catabolic state may lead to extravasation of fluid to the subcutaneous tissue, resulting in peripheral oedema. This may be exacerbated by the increased capillary permeability associated with chronic hyperglycaemia (11). Renal tubular sodium reabsorption is enhanced by insulin therapy via stimulating the Na^+^/K^+^-ATPase as well as the expression of Na+/H+ exchanger 3 in the proximal tubule (12, 13). Transient inappropriate hyperaldosteronism has also been suggested to contribute to the fluid retention (12). In our cases, normal plasma aldosterone/renin ratio was accompanied by significant natriuresis, leading to recovery of the abnormal sodium handling in the renal tubules. Interestingly, many of the described patients with insulin-induced oedema were substantially underweight, with the most dramatic presentation occurring in the underweight patients (14), a finding similar to our two cases. Our patients were thin adolescents, who had considerable weight loss before admission. In several patients it was reported that loss of albumin from the circulation due to increased transcapillary leakage probably contributed to the formation of oedema and the decreased serum albumin, but was not severe enough to account for the magnitude of oedema (15). Similar findings were true for our patients. Cases with normal serum albumin have also been reported. Clinically, insulin oedema may present with a spectrum of severity from mild pedal oedema to frank anasarca. Pleural effusions have uncommonly been reported, although some of these patients were elderly and may have had pre-existing cardiac disease. Rarely, the oedema extended from peripheral tissues to serosal cavities with ascites and cardiac failure (14, 16, 17).

In patients with insulin oedema, fluid and salt restriction should be implemented and this may be all that is necessary. Diuretic therapy may be indicated in more severe decompensated cases. Administration of an aldosterone antagonist such as spironolactone may be considered from a pathophysiological point of view in the presence of inappropriate hyperaldosteronism; however, the treatment with other diuretics appears to be also effective (12). In most instances no therapy is needed and spontaneous recovery is noted, as was the case in our patients.
